# Optimization of melanin pigment production from the halotolerant black yeast *Hortaea werneckii* AS1 isolated from solar salter in Alexandria

**DOI:** 10.1186/s12866-022-02505-1

**Published:** 2022-04-08

**Authors:** Asmaa Elsayis, Sahar W. M. Hassan, Khaled M. Ghanem, Heba Khairy

**Affiliations:** 1 National Institute of Oceanography and Fisheries (NIOF), Cairo, Egypt; 2 Department of Botany and Microbiology, Faculty of Science, Alexandria University, Alexandria, Egypt

**Keywords:** Melanin, Black yeast, *Hortaea werneckii*, Plackett-Burman design, Box-Behnken

## Abstract

**Background:**

Melanins are one of the magnificent natural pigments synthesized by a wide range of microorganisms including different species of fungi and bacteria. Marine black yeasts appear to be potential prospects for the synthesis of natural melanin pigment. As a result, the goal of this research was to isolate a marine black yeast melanin-producing strain and improve the culturing conditions in order to maximize the yield of such a valuable pigment.

**Results:**

Among five locally isolated black yeast strains, the only one that demonstrated a potent remarkable melanin pigment production was identified using ITS rDNA as *Hortaea werneckii* AS1. The extracted pigment’s physiochemical characterization and analytical investigation with Ultraviolet-Visible (UV) spectrophotometry, Fourier Transform-Infrared spectroscopy (FTIR), and Scanning Electron Microscope (SEM) confirmed its nature as a melanin pigment. The data obtained from the polynomial model’s maximum point suggested that CaCl_2_, 1.125 g/L; trace element, 0.25 ml/L; and a culture volume 225 mL/500 mL at their optimal values were the critical three elements impacting melanin production. In comparison with the baseline settings, the response surface methodology (RSM) optimization approach resulted in a 2.0 - fold improvement in melanin output.

**Conclusions:**

A maximum melanin yield of 0.938 g/L proved the halotolerant *H. werneckii* AS1 potentiality as a source for natural melanin pigment synthesis ‘when compared to some relevant black yeast strains’ and hence, facilitating its incorporation in a variety of pharmaceutical and environmental applications.

**Supplementary Information:**

The online version contains supplementary material available at 10.1186/s12866-022-02505-1.

## Background

Melanin pigments are a class of high molecular weight metabolites displayed in dark colors ranging from brown to black, which is synthetized from phenolic or indolic compounds and often present in complexes with proteins or carbohydrates [1, 2]. Principally, their formation backs to either the auto-oxidation process of the phenolic compounds in the medium or enzymatically through the action of tyrosinase and polyketide synthase as key enzymes in melanogenesis [3]. Marine fungi include a particular group of black yeasts capable of producing these valuable coloring metabolites as they have a fundamental role in standing the extreme environmental conditions [4–6].

Black yeasts as polymorphic fungi showed yeast-like, filamentous, and meristematic growth. Their hyphal growth is demonstrated on solidified media, predominantly [7, 8]. Melanin production is suggested to contribute to the widespread distribution of black fungi in extreme environments as one of their mechanisms for survival and/or adaptation under harsh conditions [9], since melanin provides the fungal cells with high level of protection in the face of various environmental stresses, mainly UV radiation [10], oxidative stress [11], the toxic effect of heavy metals, and high salt concentrations [12–14]. The melanin producing ascomycetous fungi in the order Dothideales or Capnodiales are considered the predominant fungal species in salterns, for example; *Hortaea werneckii*, *Aureobasidium pullulans*, *Trimmatostroma salinum*, *Neophaeotheca triangularis*, in addition to phylogenetically related species of *Cladosporium *[15–17].

*H. werneckii* species among saltern’s black yeasts, has received the most attention. It is an extremely halotolerant species [17] that withstand salinities (NaCl concentrations) in the range of 0% to saturation 32% (w/v), with optimal growth at 6-10% (w/v) [18–21]. In fact, it was spotted in a variety of salty habitats, including seawater, sea sponges, mangrove plants, as well as salted foods [22, 23]. Despite the fact that *H. werneckii* has been documented to synthesize dihydroxynaphthalene (DHN) melanin under both saline and non-saline conditions of growth [24], melanin granules were salt-based distributed in the outer part of the melanized cell wall, allowing *H. werneckii to* successfully minimize the permeability for the main compatible solute glycerol through reducing pores size in the cell wall. This mechanism could be one of the characteristics that contribute to its tolerance to a wide range of salt concentrations [25, 26].

Distinctive properties of melanins allow their participation in a wide range of applications among various fields. First, medical applications include antimicrobial, antiviral, antitumor, antioxidant, and anti-inflammation [27–30]. Second, pharmaceutical and cosmetic applications because of their capacity to absorb broad spectrum electromagnetic radiation ranging from visible light and ultraviolet radiation to the X-rays [31–33]. Third, environmental application in bioremediation of contaminated sites backing to their capacity of binding heavy metals and radionuclides [34].

Due to their useful proprieties and ability to be used in a variety of applications, there is a growing demand for melanin production [35]. Furthermore, marine species as a source for the production of microbial melanin pigment, in particular, is an appealing option for both researchers and industries due to a variety of criteria such as being safe, easily degradable, and eco-friendly without causing adverse side effects [36]. From this point of view, our work aimed first for isolation of promising melanin-producing strain from Egyptian marine habitat, followed by, characterization and enhancement of melanin pigment production using a mathematical technique for conditions optimization in order to reach the maximum melanin yield.

## Results

### Screening for a promising melanin pigment producing isolate

Five melanized isolates (AS1 to AS5) were recovered from local marine habitat using various sediment and water samples. Two isolates (AS3 and AS4) were obtained from water samples, while (AS1, AS2, and AS5) were recovered from sediments. All of the obtained isolates appeared to have dark brown to black colored colonies on SDA plates supplemented with L-tyrosine and 10% sodium chloride and were considered as potential melanin pigment producers. Melanin production of the five isolates was investigated under both shaking (180 rpm) and static conditions, results revealed that shaking is a must for growth and melanin production by all of them. Furthermore, AS1 isolate (Additional file 1: Fig. 1) among all of them showed the highest yield in both biomass and melanin recording 9.3 g/L and 0.420 g/L, respectively (Additional file 1: Fig. 2). Therefore, AS1 was used for further experiments.

### Molecular identification & phylogenetic relationship of the potent isolate

The black yeast isolate AS1 was identified using Internal Transcribed Spacer (ITS) sequencing, resulting in 536 base pairs of sequencing data. This sequence was compared to rDNA gene sequences present in the NCBI database using the BLAST search computer-based program. BLAST analysis confirmed 99 percent similarity of strain AS1 to the species *Hortaea werneckii* AUMC 10270 (KX233858). The nucleotide sequence was deposited in the GenBank database with the accession number (MW187022). In addition, the isolate was deposited in Assuit University Mycological Center with the number (AUMC 14501).

**Fig. 1:**
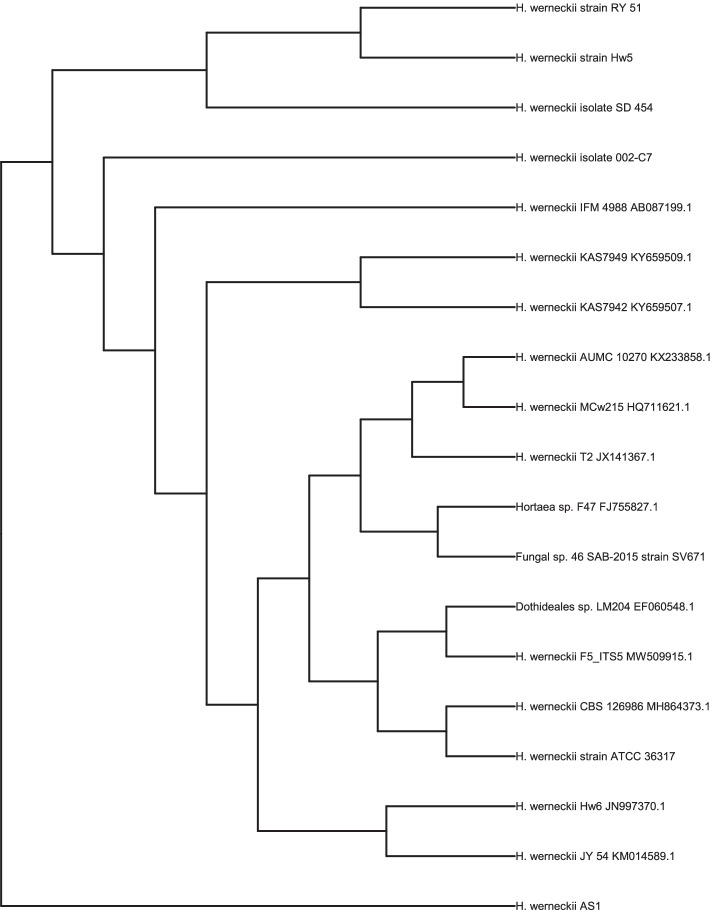
Phylogenetic tree based on ITS sequences showing the position of strain for *Hortaea werneckii* AS1. These phylogenetic relationships were identified by BioEdit sequence alignment editor (version 7.2.5)

The phylogenetic position of *H. werneckii* AS1 and the closely related relatives were analyzed using neighbor-joining dendrogram as shown in (Fig. 1).

### Melanin characterization

#### Physiochemical characterization

Melanin's unique solubility and reactivity properties allowed the usage of conventional physicochemical tests as a primary step towards the identification and characterization of the produced pigment. *H. werneckii* AS1 extracted dark pigment was chemically characterized and results revealed that it was insoluble in either water or most of the organic solvents tested (butanol, chloroform, ethyl acetate, and hexane). On the other hand, the pigment showed a slight solubility in both ethanol and methanol solvents. Also, the pigment was observed to be radially soluble only in a sodium hydroxide (1N) solution, and after vigorous shaking in dimethyl sulfoxide (DMSO). Moreover, it exhibited precipitation in 1% ferric chloride solution and decolorized by hydrogen peroxide 30% (v/v) (Additional file 1: Fig. 3). The solubility tests of the melanin standard were performed in parallel with the extracted pigment and recorded the same results.

#### Spectrophotometric analysis

**Fig. 2:**
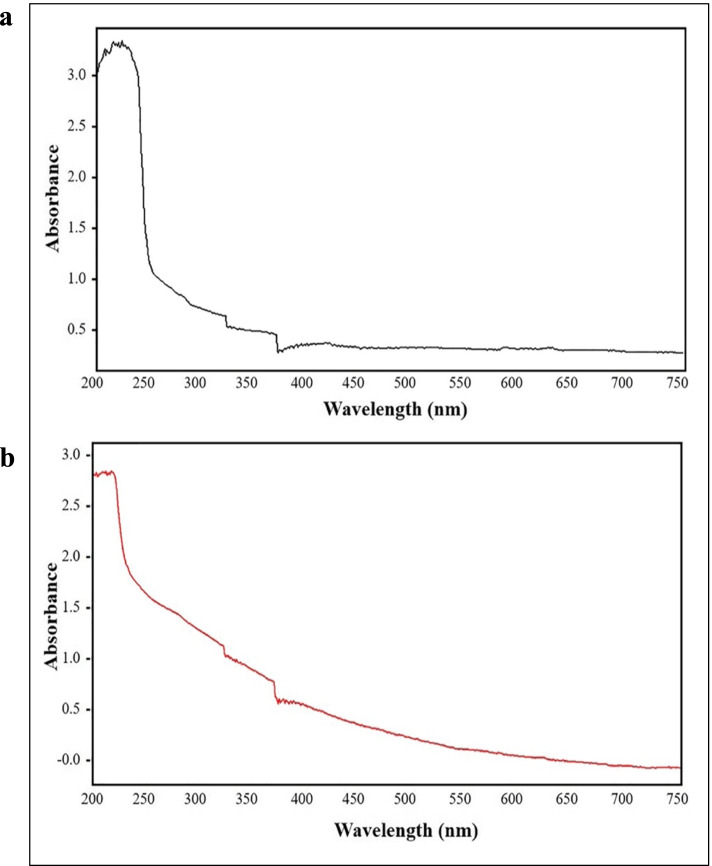
UV-visible spectral properties of (**a**) *Hortaea werneckii* AS1 pigment and (**b**) standard melanin

The extracted pigment in parallel with melanin standard (Sigma Aldrich) were spectrophotometrically analyzed in a wavelength range of (200-800 nm) (Fig. 2a, b). A similar UV-visible absorption spectrum pattern was obtained by both the biosynthesized and synthetic melanin, revealing that absorption peak was at UV region and declined towards the visible region with maximum points of absorption at 240 nm and 219 nm, respectively.

#### Fourier transform-infrared (FT-IR) analysis

**Fig. 3:**
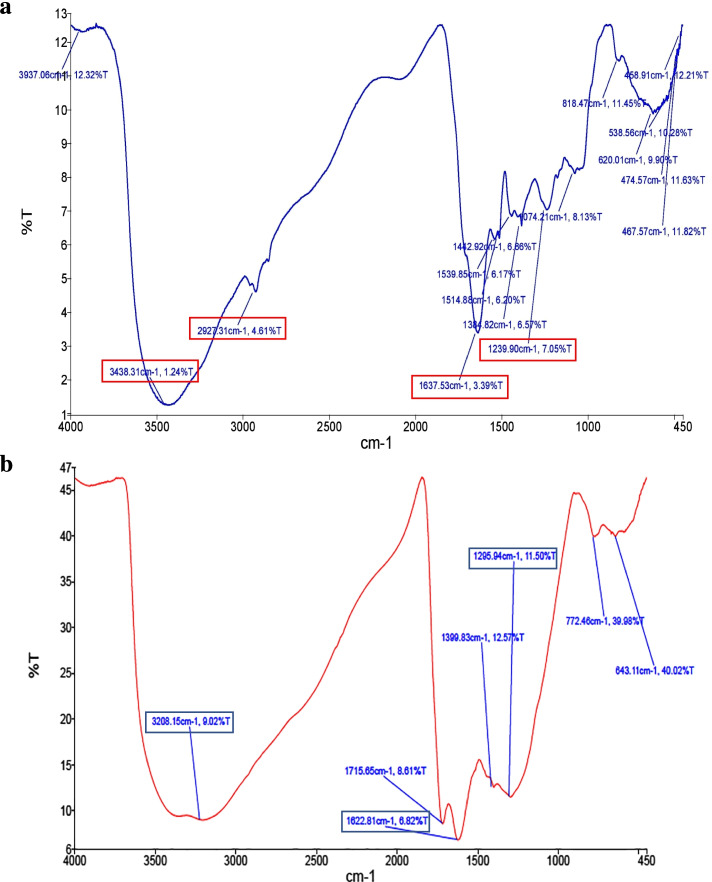
FTIR spectrum of (**a**) *Hortaea werneckii* AS1 pigment and (**b**) standard melanin showing number of the pigment characteristic peaks

The obtained FTIR spectrum of the pigment derived from *H. wernekii* AS1 (Fig. 3a) showed a number of significant peaks near 3438.31 cm^-1^, 2927.31 cm^-1^, 1637.53 cm^-1^, and 1239.90 cm^-1^. The result was compared to a melanin standard acquired from Sigma Aldrich (Fig. 3b). The most noticeable difference between *H. werneckii* AS1 pigment and synthetic melanin standard is the peak at 2927 cm^-1^, which also implies the presence of a considerable amount of aliphatic groups in the investigated melanin structure.

#### Elemental analysis

The amounts of nitrogen, carbon, hydrogen, and sulphur in both the extracted pigment and melanin standard were detected through the elemental analysis. The elemental structure of extracted pigment showed higher percentages of hydrogen and sulphur (5.644% and 0.877%) in comparison with synthetic melanin (3.196% and 0.582%), respectively. On the other hand, the extracted pigment’s carbon and nitrogen contents (43.35% and 5.48%) were almost similar to synthetic melanin values (47.82% and 5.78%), respectively.

#### Scanning electron microscope of the derived melanin pigment

**Fig. 4:**
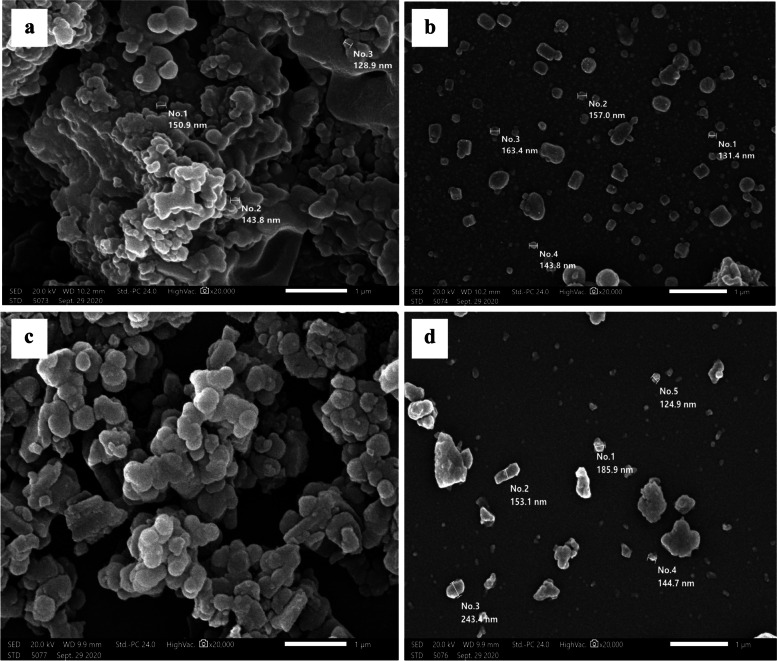
SEM analysis of melanin (**a**) overall view of *Hortaea werneckii* AS1 pigment, (**b**) focused view showing size of *Hortaea werneckii* AS1 melanin crystals (**c**) overall view of synthetic melanin and (**d)** size of synthetic melanin crystals

The morphological characterization and structural arrangement of the extracted pigment in parallel with synthetic melanin have been analyzed by SEM and revealed that *H. werneckii* AS1 pigment has a definite crystal shape besides, structures appear like yeast cells (Fig. 4a, c). The extracted pigment particle’s average size lies between (130-160 nm) (Fig. 4b), with a slight difference from the synthetic standard melanin range (125-240 nm) (Fig. 4d).

### Optimization of melanin pigment production

#### Effect of incubation period on growth and melanin production

The incubation period’s impact (1-11 days) over several criteria like dry weight and mainly melanin yield was studied. As illustrated in (Additional file 1: Fig. 4), the biomass dry weight and maximum melanin yield were achieved in the 9^th^ and 10^th^ days of incubation with about 11.4 g/L and 0.44 g/L, respectively, then the numbers started in decreasing gradually. Observation of the time course of melanin production and incubation period showed that the yield level increased after the 5^th^ day and reached its maximum on the 10^th^ day (stationary phase of growth). Similar results were noticed regarding the dry weight with a slight difference in having the maximum yield in the 9^th^ day and started to decrease afterward.

#### Different sodium chloride concentrations effect on the isolate’s growth and melanin synthesis

The impact of various concentrations of sodium chloride on *H. werneckii* AS1 dry weight and melanin productivity was tested (Additional file 1: Fig. 5). Melanin production dropped as sodium chloride concentrations increased, with the highest output (0.469 g/L) recorded at 3.5% salinity (seawater salinity) and the lowest yield (0.073 g/L) observed at 20% salinity in a medium prepared with sea water. The optimum growth rate and biomass productivity of *H. werneckii* AS1 were at about 13.5% salinity with a 13.8 g/L yield.

#### Optimization of different medium components by Plackett- Burman (PB) design of experiment

In order to assess the effect of several influences on biomass and melanin synthesis in submerged cultures, Plackett-Burman experimental design was employed. Elven variables were examined in PB experiments and the experimental results indicated as showed in Table 1 that the most productive trial was (trial 6) with the highest melanin productivity of about (0.876 g/L). For each independent variable, the main effect as well as t-values, were calculated (Table 1).

**Table 1:** The applied Plackett–Burman experimental design and the statistical analysis for elven cultural variables for the production of melanin pigment by *H. werneckii* AS1

Trial	Factor symbol											Response
	Glucose (g/L)	KNO_**3**_ (g/L)	K_**2**_HPO_**4**_ (g/L)	CaCl_**2**_ (g/L)	MgSO_**4**_ (g/L)	Tyrosine (g\L)	Trace element (mL/L)	pH	Culture age (day)	Inoculum size (mL/L)	Volume (mL/500mL)	Melanin (g/L)
**1**	−1[10]	−1[7.5]	−1[1.25]	+1[0.75]	+1[1.5]	+1[1.5]	−1[0.5]	+1[6.5]	+1[10]	−1[0.5]	+1[150]	0.538
**2**	−1[10]	−1[7.5]	+1[3.75]	+1[0.75]	+1[1.5]	−1[0.5]	+1[1.5]	+1[6.5]	−1[4]	+1[1.5]	−1[50]	0.217
**3**	−1[10]	+1[22.5]	−1[1.25]	−1[0.25]	−1[0.5]	+1[1.5]	+1[1.5]	+1[6.5]	−1[4]	+1[1.5]	+1[150]	0.327
**4**	+1[30]	+1[22.5]	−1[1.25]	+1[0.75]	−1[0.5]	−1[0.5]	−1[0.5]	+1[6.5]	+1[10]	+1[1.5]	−1[50]	0.531
**5**	−1[10]	+1[22.5]	+1[3.75]	−1[0.25]	+1[1.5]	−1[0.5]	−1[0.5]	−1[4.5]	+1[10]	+1[1.5]	+1[150]	0.340
**6**	+1[30]	−1[7.5]	+1[3.75]	+1[0.75]	−1[0.5]	+1[1.5]	−1[0.5]	−1[4.5]	−1[4]	+1[1.5]	+1[150]	**0.876**
**7**	+1[30]	−1[7.5]	+1[3.75]	−1[0.25]	−1[0.5]	−1[0.5]	+1[1.5]	+1[6.5]	+1[10]	−1[0.5]	+1[150]	0.219
**8**	+1[30]	−1[7.5]	−1[1.25]	−1[0.25]	+1[1.5]	+1[1.5]	+1[1.5]	−1[4.5]	+1[10]	+1[1.5]	−1[50]	0.029
**9**	+1[30]	+1[22.5]	+1[3.75]	−1[0.25]	+1[1.5]	+1[1.5]	−1[0.5]	+1[6.5]	−1[4]	−1[0.5]	−1[50]	0.046
**10**	−1[10]	−1[7.5]	−1[1.25]	−1[0.25]	−1[0.5]	−1[0.5]	−1[0.5]	−1[4.5]	−1[4]	−1[0.5]	−1[50]	0.091
**11**	−1[10]	+1[22.5]	+1[3.75]	+1[0.75]	−1[0.5]	+1[1.5]	+1[1.5]	−1[4.5]	+1[10]	−1[0.5]	−1[50]	0.153
**12**	+1[30]	+1[22.5]	−1[1.25]	+1[0.75]	+1[1.5]	−1[0.5]	+1[1.5]	−1[4.5]	−1[4]	−1[0.5]	+1[150]	0.598
**13**	0[20]	0[15]	0[2.5]	0[0.5]	0[1]	0[1]	0[1]	0[5.5]	0[7]	0[1]	0[100]	0.431
**Main effect**	0.11	0.004	-0.044	0.31	-0.07	-0.005	-0.15	-0.035	-0.06	0.11	0.31	
***t*-value**	0.69	0.02	0.28	2.53	0.46	0.02	1.0	0.22	0.37	0.73	2.47	

*t*-value significant at the 1% level = 3.70

*t*-value significant at the 5% level = 2.446

*t*-value significant at the 10% level = 1.94

*t*-value significant at the 20% level =1.372

Standard t-values are obtained from Statistical Methods [37]

According to the obtained results, calcium chloride, culture volume and trace element were the most significant independent variable that affects melanin production by *H. werneckii* AS1 with a *t*-values of 2.53, 2.47 and 1.0, respectively. Also, the main effect results demonstrated that a positive level of calcium chloride and culture volume, in addition to the negative level of trace elements aided in the melanin formation.

An experiment of verification was used to compare the estimated ideal levels of independent variables and the baseline settings to assess the perfection of the practiced Plackett-Burman statistical design. Results indicated that melanin productivity increased to 0.509 g/L with a 1.23-fold increase in comparison with the product obtained under the basal conditions.

#### Growth parameters optimization using Box Behnken (BB) design

The most important independent factors suggested by the Plackett-Burman design were investigated using Box Behnken (BB) design. The design matrix and the concentration of pigment response (g/L) of each trial were illustrated in (Table 2). The determination coefficient R2 for pigment production was 0.853 for this experiment, showing a remarkable degree of relation among experimental and predicted values. A polynomial function of second-order was fitted to the response results of the experiment (nonlinear optimization algorithm) to anticipate the ideal variable values;

$$\left(\mathrm{Y}=0.64+0.13625{\mathrm{X}}_1+0.015{\mathrm{X}}_2+0.22875{\mathrm{X}}_3-0.045{\mathrm{X}}_1{\mathrm{X}}_2-0.0825{\mathrm{X}}_1{\mathrm{X}}_3+0.015{\mathrm{X}}_2{\mathrm{X}}_3+0.09125{{\mathrm{X}}_1}^2+0.02375{{\mathrm{X}}_2}^2-0.05875{{\mathrm{X}}_3}^2\right)$$

**Table 2:** Box-Behnken factorial experimental design for melanin pigment production by *Hortaea werneckii* AS1

Runs	Experimental parameters			Response (g/L)
	CaCl2 (g/L)	Trace element (mL/L)	Culture volume (mL/500mL)	
1	0[0.75]	0[0.5]	0[150]	0.58
2	-1[0.375]	1[0.75]	0[150]	0.59
3	0[0.75]	-1[0.25]	-1[75]	0.44
4	1[1.125]	0[0.5]	-1[75]	0.51
5	0[0.75]	1[0.75]	-1[75]	0.41
6	1[1.125]	-1[0.25]	0[150]	1.01
7	-1[0.375]	-1[0.25]	0[150]	0.44
8	0[0.75]	1[0.75]	1[225]	0.8
9	1[1.125]	0[0.5]	1[225]	0.9
10	0[0.75]	0[0.5]	0[150]	0.69
11	0[0.75]	-1[0.25]	1[225]	0.77
12	-1[0.375]	0[0.5]	-1[75]	0.28
13	-1[0.375]	0[0.5]	1[225]	1
14	1[1.125]	1[0.75]	0[150]	0.98
15	0[0.75]	0[0.5]	0[150]	0.65

The optimal degree of the 3 factors as determined from the polynomial model’s maximum point, were calculated by applying the *solver* function of the (Microsoft Excel 2019) tools and detected to be (calcium chloride, 1.125 g; Trace element, 0.25 mL, and Culture volume 225 mL) with a predicted melanin yield of 0.994 g/L.

**Fig. 5:**
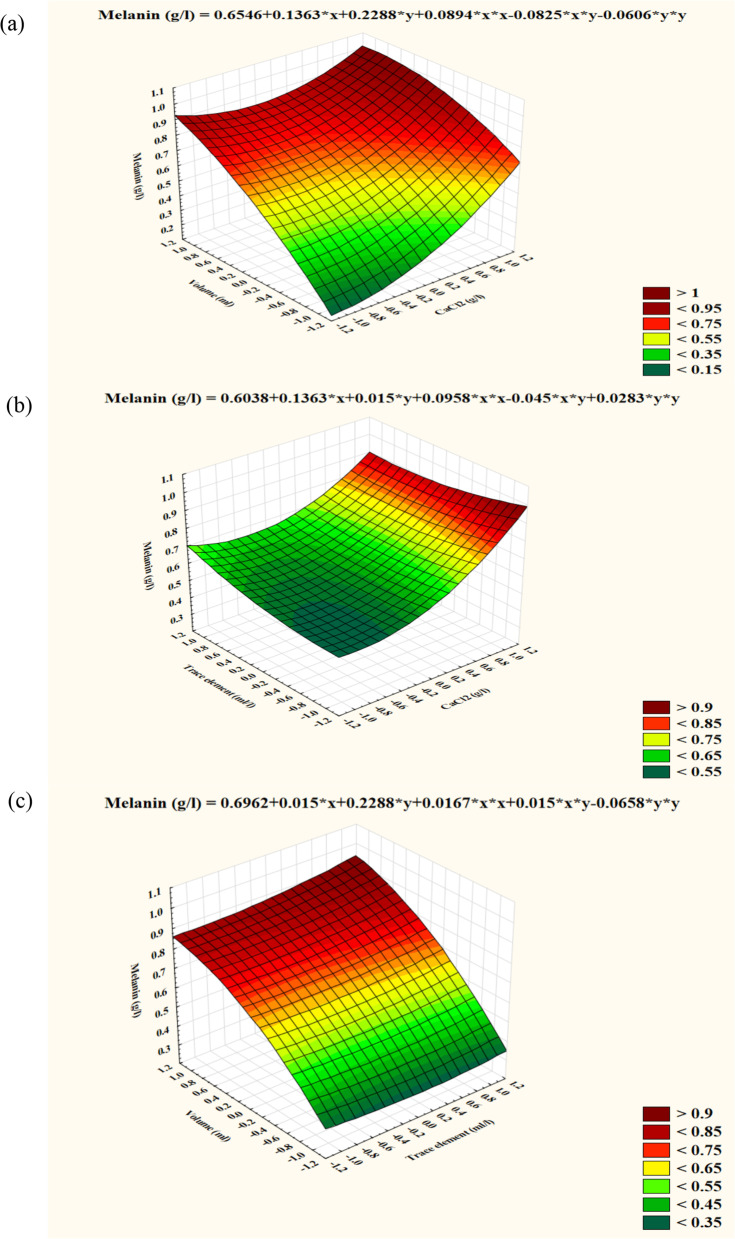
Three-dimensional surface plots showing the relationships between the tested significant variables and pigment formation by *Hortaea werneckii* AS1 and the optimal levels of the three factors as obtained from the maximum of the polynomial model. The three figures indicate (**a**) the interaction between CaCl_2_ and culture volume, (**b**) the interaction between CaCl_2_ and trace element and (**c**) the interaction between trace element and culture volume

A three-dimensional (3D) response surface curves by STATISTICA 14.0 software were plotted, in order to reveal the interaction effects and ideal grade of the variables (Fig. 5a, b and c). As illustrated in (Fig. 5a), an interaction between calcium chloride and culture volume at their high values was recognized where, the maximum level of melanin synthesis was documented. On the other hand, the curve had its maximum response near the trace element low values (Fig. 5b). Previously mentioned results indicated that high calcium chloride with minimum trace element values were supportive in increasing melanin productivity by *H. werneckii* AS1. Besides, in (Fig. 5c) curve had its maximum along the trace element axis near its lower levels indicating that high culture volume with low trace element concentrations gave the highest amount of melanin.

As a result, the polynomial model’s maximum point determined the optimal values of the three factors and were recorded as calcium chloride, 1.125 (g/L); trace element, 0.25 (mL/L), and a culture volume 225 mL.

The experimentally validated optimal conditions from the optimization experiment were put in comparison with the data calculated from the model. The maximum amount of melanin produced (0.938 g/L) was obtained, which was slightly lower than the model’s predicted value (0.994 g/L). The model's validity was demonstrated by the tight corelation between experimental and predicted values.

Asecond verification experiment was performed to compare the productivity of the basal and the optimized medium, and data represented that melanin productivity in the optimized one was about 1.45 fold higher than the basal medium (0.646 g/L).

From previously illustrated data it can be predicted that in order to obtain the highest yield of melanin pigment by *H. werneckii* AS1, the medium needs to be formulated as following (g/L): glucose, 30; KNO_3_, 22.5; K_2_HPO_4_, 1.25; MgSO_4_.7H_2_O, 0.5; CaCl_2_.2H_2_O, 1.125; tyrosine, 0.5; trace element, 0.25 ml; culture age, 4 days; inoculum size, 1.5ml; culture volume, 225 adjusted to pH 4.5 and incubation period 10 days at 30 ^o^C with agitation speed 180 rpm.

Previously mentioned results, revealed that *H. werneckii* AS1 is a promising locally isolated black yeast strain proved to synthesize melanin pigment in a considerable amount.

## Discussion

*Hortaea werneckii* strain isolated in our study from solar salter (as a biological source of melanin pigment), has been documented as an extremely halotolerant fungus [38], and was recurrently isolated from natural hypersaline habitats all over the world [39]. In accordance, A three black yeast strains (EGYNDA08, EGYNDA16, and EGYNDA90) that was identified as *H. werneckii* were isolated from the Egyptian offshore salt marshes [40]. In addition, under optimal conditions, *H. werneckii* EGYNDA08 was able to tolerate NaCl concentrations of up to 25% and significantly produce a dark brown melanin pigment of up to 228 mg/L [1]. Also, an extremophilic black yeast *H. werneckii* strain was reported to be isolated from decayed leaves from the Red Sea mangroves [3].

The direct effect of agitation on *H. werneckii* productivity of melanin may back to the fact that most aerobic microorganism’s processes are governed by the rate of oxygen transfer from air to liquid cultivation media; thus, shaking frequency is a critical factor in increasing oxygen circulation affecting growth and pigment production [41, 42].

*H. werneckii* AS1 had the maximum melanin synthesis of 0.420 g/L when shaked at 180 rpm. In parallel with this findings, effect of agitation on *Aspergillus oryzae* growth, tyrosinase biosynthesis, and melanin pigment production has been documented with an optimum agitation rate of about 200 rpm resulted in promoting melanin pigment biosynthesis to about (0.258 mg/mL) [43]. Moreover, Thaira et al. [42] found that the highest melanin production by *Pseudomonas stutzeri* strain HMGM-7 was found to be (248 mg/L) and was also positively affected by agitation speed of about 200 rpm. They stated that higher and lower shaking frequencies (250 and 100 rpm) tend to reduce melanin production and biomass, respectively. Insufficient aeration during low shaking frequencies (100 rpm), may be the reason for the decrease in biomass production. On the other hand, shaking frequency as high as (250 rpm) negatively affected the growth and melanin biosynthesis because of generating excessive foam which resulted in reduced oxygen transfer rate.

Melanins are negatively charged, stable pigments with low solubility in both aqueous and most organic and inorganic solvents [44, 45]. Solubility characteristics of the extracted pigment from *H. werneckii* AS1 were identical to standard melanin results, as well as, similar to properties of melanins reported previously from other microorganisms [1, 43. 46]. Similar to our data, the extracellular melanin pigment synthesized by marine *Nocardiopsis* spp. were found to be insoluble in ethyl acetate and chloroform but were soluble in dimethylsulfoxide and alkaline water (pH 10) [47].

According to reports on the UV properties of melanin, the alkali solutions optimal absorption wavelength among the majority of melanin types lies in the range of 196-300 nm, depending on its origin. In addition, alkaline solutions of melanin exhibit a high UV region optical absorbance, which gradually diminish as it migrates to longer wavelengths. Previously mentioned unique melanin behavior may back to a kind of melanin’s conjugated molecule’s complex structure which engage and disperse UV light photons [46, 48].

The UV absorption characteristics of *H. werneckii* AS1 melanin with maximum point at 240 nm were consistent with the UV spectra of melanins from various fungi and bacteria. Melanin synthesized by Antarctic bacterium *Lysobacter oligotrophicus* strain 107-E2^T^ and *H. werneckii* EGYNDA08 exhibited characteristic peaks with strong absorbance in the UV region detected in the range of 200-300 nm [1, 44]. Following the same manner, melanin pigment extracted from *Cryptococcus rajasthanensis* KY627764 showed significant absorbance peaks at 244 nm and 220 nm, respectively in comparison with melanin standard [49].

In addition, the IR spectrum of *H. wernekii* AS1 derived pigment showed a number of significant peaks near 3438.31 cm^-1^, 2927.31 cm^-1^, 1637.53 cm^-1^, and 1239.90 cm^-1^. The first peak revealed the presence of the (-OH) group, the broadening of the band may be justified by linkage of the amino group (-NH) with the (-OH) group through hydrogen bonding [49]. While the second peak indicates the stretches of aliphatic (C-H groups). Moreover, the aromatic ring (C=C) stretching was linked to the third one. The last one represented the phenolic (-OH) groups stretching vibration. These FTIR features have a close similarity to the typical structure of melanin [45]. Characteristic properties of the IR spectrum of *H. werneckii* AS1 pigment were similar to melanin pigment from various reported microorganisms as *Klebsiella* sp. GSK [50], *Inonotus hispidus* mushroom [46], and *H. werneckii* EGYNDA08 [1]. In accordance to the above-mentioned findings, Infrared (IR) spectrum of melanin from *H. werneckii,* halophilic black yeast, revealed characteristic peaks near 3,435 cm^-1^, 2,931 cm^-1^, 1,633 cm^-1^, and 1,267 cm^-1^, respectively [51]. Furthermore, melanin pigments from *Cryomyces antarcticus*, a black fungus, displayed two remarkable peaks around 2917 and 2843 cm^−1^. Also, as indicated in this work, those peaks were not detected in the conventional DOPA melanin spectra used for comparison [52]. Mbonyiryivuze et al. [53], however, ascribed those peaks to DOPA melanin.

In general, fungal DOPA-melanin are derived from the oxidation of L-dopa or L-tyrosine. They are remarked by having about 5.1−9% nitrogen and 0−1% sulfur of their elemental composition, which means that they almost lacking sulfur in their structure [30, 54]. Another main fungal melanin type is DHN-melanins characterized by the absence of nitrogen in their structure [30, 45, 46, 55]. From previously mentioned findings and due to acceptable CHN content (43.35%, 5.644%, and 5.48%, respectively) and the concentration of sulfur (0.877%) that is very low, we suspect that the examined pigment may be eumelanin. According to Chen et al. [56] the small quantity of sulfur may be retained during the purification steps or a small amount of a sulfur-containing protein attached to the melanin.

A nitrogen percentage varying from 5.1 to 6.7% with complete lacking of the sulfur element was documented for DOPA-melanin produced by *Aspergillus nidulans* strains [57]. In addition, DOPA-melanin from *Inonotus hispidus* mushroom revealed a similar nitrogen content of around 5.31% with a higher sulfur content of 2.02% [46]. *Boletus griseus*, halophilic black yeast *H. werneckii* and *Klebsiella* sp. GSK all have similar findings regarding the elemental composition of melanin pigments [51, 58, 59].

The shape and size of melanin granules vary depending on the source between (30-1000 nm) [45]. *H. werneckii* AS1 melanin was figured as definite crystals and/or yeast cells with sizes ranging from (130-160 nm). In harmony with the current investigation, Rani et al. [51] reported the melanin pigment derived from the halophilic black yeast *H. werneckii* to has a yeast resembling structure. In contrast, *Hortaea werneckii* R23 melanin was characterized to has a definite crystal shape with a defined structural order [60]. In addition, melanin pigment extracted from *Mycosphaerella fijiensis* mycelium was described as large clusters of compact spherical granules ranging in diameter between (100–300 nm) [61].

The productivity pattern of *H. werneckii* AS1 melanin showed a slow exponential phase output, which increased till obtaining the onset at the stationary phase in the 10^th^ day with about 0.44 g/L. A similar behavior was documented in previous studies for other microorganisms as *Aspergillus bridgeri*, *Actinoalloteichus* sp, and *Yarrowia lipolytica* [32, 27, 35]. On the contrary, melanin production by the halophilic black yeast *H. werneckii* showed incubation periods shorter than 10 days and the maximum yield of crude melanin was 5.60 g/L extracted on the 6^th^ day [51]. A decrease in melanin yield may be due to photodegradation of the pigment or by the organism itself since melanin is a photo-protective polymer [43].

Regarding the growth behavior of our isolate under a range of different salinities, it followed the characteristic pattern of growing without salt, as well as in a salt solution as high as (4 M) but in a slower growth rate. The findings of Saleh et al. [1] on *H. werneckii* EGYNDA08 are consistent with the previously mentioned results since they have documented that their isolate could survive salt concentrations up to 25%, nearly saturated salt concentration, and even under the entire lack of salt. They also, observed that melanin concentration decreased by increasing salinity and they justify that with the effect of growth medium on melanization. Ultrastructural studies on *H. werneckii* melanin revealed that at 0-0.85 M sodium chloride, a continuous thin coating of melanin granules was constructed, which can develop into a shield-like firm layer of melanin granules in the outside section of the cell wall and can also stretch into the outer layer of the cell wall at ideal salinity [26]. However, when grown under high salinities of 1.7 and 3.4 M sodium chloride, an incoherent layer of melanin granules was formed. These facts may clarify the decrease in melanin production by increasing NaCl concentration due to a change in its deposition structure in the cell wall.

Researches on the production of melanin from melanogenic microorganisms revealed that there is no ubiquitous media or cultivation conditions for propagating them. Moreover, the composition and ratio of each component vary regarding the isolate [62]. Hence, applying the statistical optimization designs can aid significantly in increasing the melanin yield by detecting the main factors affecting the process, besides the impact of the interaction between those valuable elements. In the present study, this technique resulted in a 2.26-fold increase in melanin yield when compared to the outcome before the whole optimization steps.

From the baseline settings, the Plackett–Burman experiment raised melanin output to 0.509 g/L with a 1.23-fold enhancement. In a parallel study, it was detected that after implement of Plackett–Burman design, the final melanin yield processed by *Streptomyces glaucescens* NEAE-H was about 19.17 μg melanin/0.1 mL of medium with a 2.24-fold increase in productivity over the basal conditions yield of 8.57 μg/0.1 mL of medium. In addition, the applied Placket-Burman experiments demonstrated that the incubation duration, protease-peptone, in addition to ferric ammonium citrate were the most valuable independent factors exerting an effect on melanin synthesis [48].

After the Plackett–Burman step, Box–Behnken design was implemented to boost melanin productivity to 0.938 g/L by improving the level and interactions between calcium chloride, trace element, and culture volume as the critical factors. In consistence with our protocol, optimization of melanin production by *Aspergillus fumigatus* AFGRD105 was applied through a 12-run of Plackett–Burman experiment for estimating the essential parameters, followed by Box–Behnken design optimization of relevant factors. Temperature, moisture, and sodium dihydrogen phosphate concentration have been mentioned to be the optimum variables. This optimization protocol resulted in a two-fold elevation in the melanin productivity from 3.4 mg/L to 6.6 mg/L [63].

Another study used the same approach to optimize *Brevundimonas* sp. SGJ productivity of melanin [64]. The best circumstances were pH 5.31, L-tyrosine 1.872 g/L, tryptone 1.440 g/L, and copper sulfate 0.0366 g/L. Melanin’s outcome with these levels was 1.227 g/L with about 3.05-fold increase over the melanin obtained prior to the optimization protocol.

Finally, the ability to get such a valuable pigment in a considerable amount from a non-hazardous natural source opens the door for its inclusion in a variety of essential applications in diverse sectors because commercially accessible melanins are obtained either chemically or by extraction from sepia, and both ways afford hurdles in terms of yields generated that aren’t suitable for large-scale applications [65]. Melanin’s appealing potential to react with various metals in a process requiring numerous engaged links between metals and the pigment’s carboxyl, hydroxyl, and amine functional groups facilitates its contribution in heavy metal bioremediation [66]. Regarding the field of human health, melanin can be used in multiple pharmaceutical, medical and cosmetic practices, including radioprotection, anticancer, antioxidant and antimicrobial [67].

## Conclusions

Melanin pigment was synthesized and extracted from the halotolerant black yeast *H. werneckii* AS1 which was isolated from Egyptian solar saltern. *H. werneckii* AS1 can withstand salt concentration up to 4 M. Physiochemical characterization and analytical analysis of the extracted pigment using UV-Visible spectroscopy, FTIR, elemental analysis, and SEM suggested it to be a eumelanin pigment. The effect of growth conditions on melanin synthesis was evaluated by the aid of statistical methods which helped in detecting the optimum level of the most important variables. Three variables calcium chloride, trace element, and culture volume were chosen for optimization of production conditions through the Box-Behnken experiment design. The optimal combination (calcium chloride, 1.125 g/L; trace element, 0.25 mL/L and a culture volume 225 mL/500mL) was obtained and the maximum melanin yield was 0.938 g/L. From the results in this study, the locally isolated *H. werneckii* AS1 can be a promising candidate for melanin production. Finally, *H. werneckii* AS1 is preserved for further studies regarding the different valuable environmental and medical applications of the produced melanin pigment.

## Methods

### Sample collection and fungal isolation

Water and sediment samples were collected from solar saltern in Egypt, exactly from Alexandria international coastal road “31°04'07.4"N 29°46'27.6"E” in December 2019. The isolation was carried out as following: one gram of sediment was suspended in 5ml of sterile sea water, then one milliliter of the dilution as well as from water samples, each were plated on Sabouraud dextrose agar (SDA) plates supplemented with L-tyrosine, 10% NaCl, and 150 mg/L chloramphenicol to avoid bacterial contamination [51, 68]. Plates were incubated at 30 °C for 3 weeks and checked at regular intervals for detecting melanized colonies, which were purified by sub-culturing on SDA made with sea water. The pure cultures were preserved in 70% glycerol and stored at -20 °C.

### Melanin production under different incubation conditions

Screening for melanin production in liquid medium was carried out in 500 mL flasks holding 100 mL of Vogel’s medium [43] prepared with seawater. Afterward, for 7 days, flasks were incubated at 30 °C in a static and shaking incubators to study and compare the impact of static and shaking modes on melanin production. For shaking condition, flasks were incubated at 180 rpm in an orbital shaker. Prior to sterilization, the pH of the culturing medium was adjusted to 5.0-5.5 [43].

### Molecular identification of the selected strain

The DNA was extracted from the potent black yeast strain culture at Assiut University’s Molecular Biology Research Unit using a Patho-gene-spin DNA/RNA extraction kit provided by Intron Biotechnology Company, Korea. After that, the fungal DNA was transported to Daejeon, South Korea, SolGent Company for performing the polymerase chain reaction (PCR) and sequencing rRNA. ITS1 and ITS4 as forward and reverse primers for the PCR reaction were added to the reaction mixture. Primers are made up of the following sequences: ITS1 (5' - TCC GTA GGT GAA CCT GCG G - 3'), and ITS4 (5'- TCC TCC GCT TAT TGA TAT GC -3'). Through the addition of ddNTPs to the mixture of the reaction, the purified PCR product (amplicons) was sequenced using the identical primers [69]. To determine DNA similarities, sequences analysis was carried out by the aid of Basic Local Alignment Search Tool (BLAST) supported by the National Center of Biotechnology Information (NCBI) website. By the help of BioEdit sequence alignment editor (version 7.2.5) [70], Phylogenetic study of ITS region sequences was performed using the Prodist-Neighbor-joining method (version 3.6a2.1).

### Melanin extraction

According to Gadd [71], melanin pigment was extracted and purified. After a week of incubation, the culture for approximately 10 minutes was centrifuged at 10,000 rpm, the supernatant was discarded and pellets were rinsed with distilled water then collected for the extraction step. Melanin was recovered from yeast biomass by autoclaving it with 1N NaOH. In order to obtain the supernatant containing the desired melanin pigment, centrifugation of the treated biomass for about 10 minutes at 8000 rpm was performed. After that, concentrated HCl was added to acidify the supernatant to pH 2 followed by a centrifugation step for 10 minutes at 10,000 rpm which resulted in precipitating the melanin pigment. The extracted melanin was then dried in a dehumidified environment.

### Melanin characterization

#### Physiochemical characterization

A modified method from that described by Thomas [72] was performed to chemically analyze and define the extracted pigment. Various inorganic and organic solvents including distilled water, 1N HCl, 1N NaOH, as well as ethanol, methanol, butanol, chloroform, dimethyl sulfoxide (DMSO), hexane, and ethyl acetate, were used to test the extracted pigment solubility pattern. Using an oxidizing agent such as H_2_O_2_, a bleaching test was carried out. In addition, 1% FeCl_3_ solution was used to test the precipitation nature of the pigment. All of these tests were done in conjunction with synthetic melanin.

#### Spectrophotometric analysis

Spectrophotometric examination of the extracted pigment was performed using UV-visible spectrophotometer over a wide wavelength range (200-800 nm). A solution of 1 N NaOH was used to dilute the pigment (1:10). As a control, a melanin standard (Sigma Aldrich; reference M8631) has been analyzed following the same steps [1].

#### Fourier transform-infrared (FT-IR) spectrum analysis

Using an IR spectrometer (PerkinElmer, USA), the extracted pigment’s FTIR spectrum was detected. The spectra were recorded in the wave region of 400-4000 cm^-1^ by using KBr pellets obtained from a mixtures of spectral grade KBr and pigment sample uniformly pressed under vacuum. The reported result was compared against a genuine standard melanin (Sigma Aldrich, reference: M8631) [31].

#### Elemental analysis

An elemental analyzer (Vario MACRO cube, Elementar, Germany) was used to detect the content of carbon, nitrogen, hydrogen, and sulphur in percent for the extracted melanin pigment. The working parameters were 0.1 percent standard deviation and 950-1200 ^o^C digesting temperatures. Final products including CO_2_, H_2_O, NO, and SO_2_ were formed from the oxidation of the analyzed products carbon, hydrogen, nitrogen and sulfur oxidize, respectively [46, 58]. The elemental composition was determined by collecting these products and estimating their weights.

#### Scanning electron microscope

The extracted pigment was covered with a coat made of gold to examine their morphological and surface structure using JEOL (JSM-IT200) scanning electron microscope (SEM) at the required magnification and an acceleration voltage of 20 kV.

### Enhancement of culture conditions for optimizing melanin pigment production

#### Incubation period effect on melanin production

The synthesis of melanin by the chosen yeast strain was evaluated in 500 mL flasks containing 100 mL of Vogel’s medium at different incubation times ranging from 5 to 10 days [73]. For each incubation period, cultures were incubated at 30 °C in an orbital shaker at 180 rpm, and biomass dry weight (g) and melanin yield (g/L) were measured.

#### Different sodium chloride concentrations effect on both growth of the isolate and melanin production

The purpose of the salinity test was to indicate how varying sodium chloride concentrations affected the production of melanin by the isolate under examination. Cells were cultured on Vogel’s medium prepared with sea water and with the addition of different NaCl concentrations 10, 20, 30, and 40% (w/v). In addition, a flask with Vogel’s medium prepared with distilled water was prepared to investigate the ability of the isolate to grow and produce melanin in the absence of NaCl. Inoculated media containing black yeast were incubated in a rotary shaker at 180 rpm for about 10 days at 30°C. The resulting melanin, at the end of the growth period and the manifestation of the black color, was derived and estimated [1, 23].

#### Optimization of different medium conditions by Plackett- Burman (PB) design experiment

Plackett-Burman experimental design was used to optimize several factors utilizing a statistical approach for maximum melanin synthesis [74]. PB design is a sort of two-level fractional factorial design that have the advantage of selecting the most important factors from a group of candidates by applying the fewest number of tests [63]. The goal of this optimization process is to figure out which medium elements have a high impact on melanin pigment formation. In this study, 12 experimental runs were used to screen 11 variables, including 7 medium component variables in addition to pH, culture age, inoculum size and culture volume. Each variable was examined at high (+) and low (-) levels, with each trial being performed twice. The following formula was used to calculate each factor’s main effect:

$$Exi=\left({Mi}_{+}- Mi-\right)/N$$

Where *Exi* is the main effect of the variable, and *Mi+* and *Mi-* are the melanin yields (mg), where the independent variable is present in high and low concentrations respectively, and *N* is the number of trials divided by two. To determine the variable significance, “Microsoft Excel 2019” was used to calculate statistical *t*-values of equal unpaired samples.

#### Optimization of growth parameters using Box Behnken (BB) design

For gaining the optimum rank of the fundamental factors that were picked up using PB experiment, BB design was conducted using independent positive variables produced after PB design. Each single variable was investigated at different three levels of low, moderate and high (−1, 0, +1). A total of 15 tests were carried out with three variables in the experimental design. The calculated response was melanin productivity (g/L) [63]. 3D surface plots were created to better understand the interactions between variables and to predict the optimum concentration of each component. The optimal medium composition for melanin synthesis was determined using a second-order polynomial model:

$$Y={\beta}_0+\Sigma {\beta}_i{X}_i+\Sigma {\beta}_{ii}{X}_{ii}+\Sigma {\beta}_{ij}{X}_{ij}$$

where *β*_*i*_ donates the regression coefficients of regression for individual factor effect, *β*_*ii*_ represents the regression coefficients for factor square effects, and *β*_*ij*_ is the regression coefficients for factors interactions. based on the analysis result(s), validation test for BB experiment were established [1]. Also, all statistical analyses and graphics were generated using STATISTICA 14.0 software and Microsoft Excel 2019.

### Nucleotides accession numbers

The consensus sequences were generated through BioEdit sequence alignment editor (version 7.2.5), and then examined by adopting the BLAST tool (National Center for Biotechnology Information (NCBI) https://www.ncbi.nlm.nih.gov/) [75]. The acquired ITS rDNA sequence was deposited in the GenBank with the number of accession MW187022. A web link for the dataset and accession number is mentioned at “Availability of data and materials” section.

## Supplementary Information

**Additional file 1.**

**Additional file 2.**

## Data Availability

All data generated or analysed during this study are included in this published article [and its supplementary information files]. Regarding the nucleotides accession number **“** The datasets generated and analysed during the current study are available in the [National Center for Biotechnology Information (NCBI) https://www.ncbi.nlm.nih.gov/**]** repository, and [The acquired 18s rRNA sequence was deposited in the GenBank with the number of accession MW187022 (https://www.ncbi.nlm.nih.gov/nuccore/MW187022)**].**
