# Supplementary material for: Optimization of melanin pigment production from the halotolerant black yeast Hortaea werneckii AS1 isolated from solar salter in Alexandria
Source: BMC Microbiol. 2022 Apr 8;22:92. doi: 10.1186/s12866-022-02505-1 (PMC8991569; doi:10.1186/s12866-022-02505-1)
Supplement: Supplementary file 1 — Additional file 1. [file 12866_2022_2505_MOESM1_ESM.pdf]

Optimization of melanin pigment production from the halotolerant black yeast *Hortaea werneckii* AS1 isolated from solar salter in Alexandria

Asmaa Elsayis[a], Sahar W. M. Hassan[a], Khaled M. Ghanem[b], Heba Khairy[b*]

[a]National Institute of Oceanography and Fisheries (NIOF), Egypt.

[b]Department of Botany and Microbiology, Faculty of Science, Alexandria University, Alexandria, Egypt.

***Corresponding author**

heba.khairy@alexu.edu.eg (Heba Khairy)

Tel: +20 127 557 7683- Fax: (002) **(03) 3921595**

Supplementary material

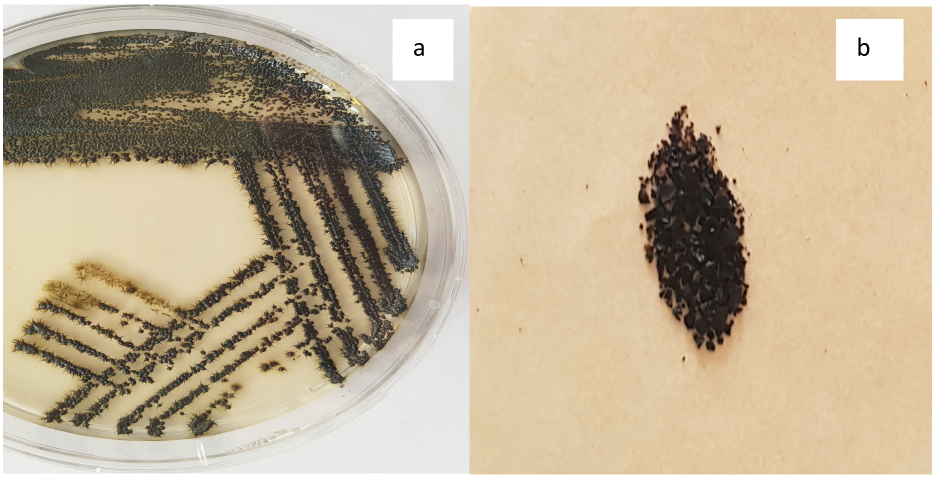

Figure 1. (a) Isolate AS1 black colonies on Sabouraud dextrose agar prepared with sea water and (b) extracted melanin

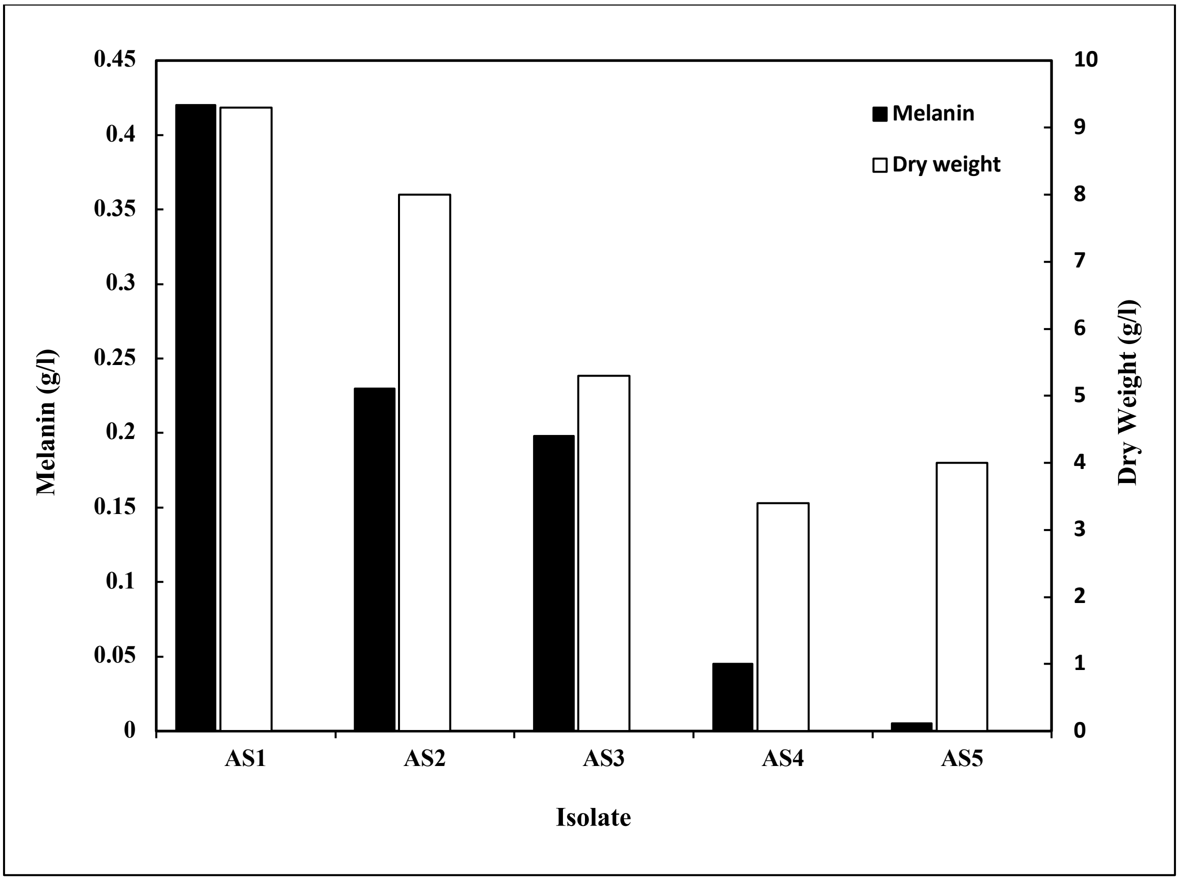

Figure 2. Biomass and melanin yield between of the isolated black yeasts.

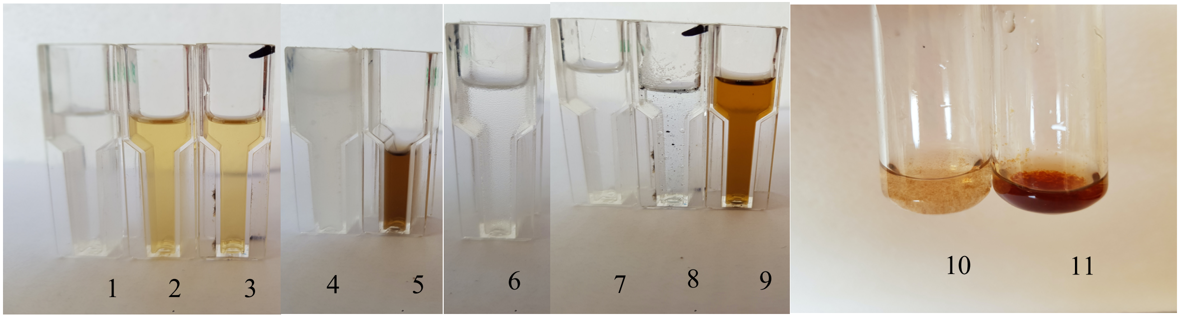

Figure 3. Solubility characteristics of extracted pigment from *H. werneckii* AS1. (1) Water, (2) ethanol, (3) methanol, (4) butanol, (5) DMSO, (6) chloroform, (7) ethyl acetate, (8) hexane, (9) 1N NaOH, (10) decolorization by H_2O_2 and (11) Brown flocculent precipitate with $FeCl_3$.

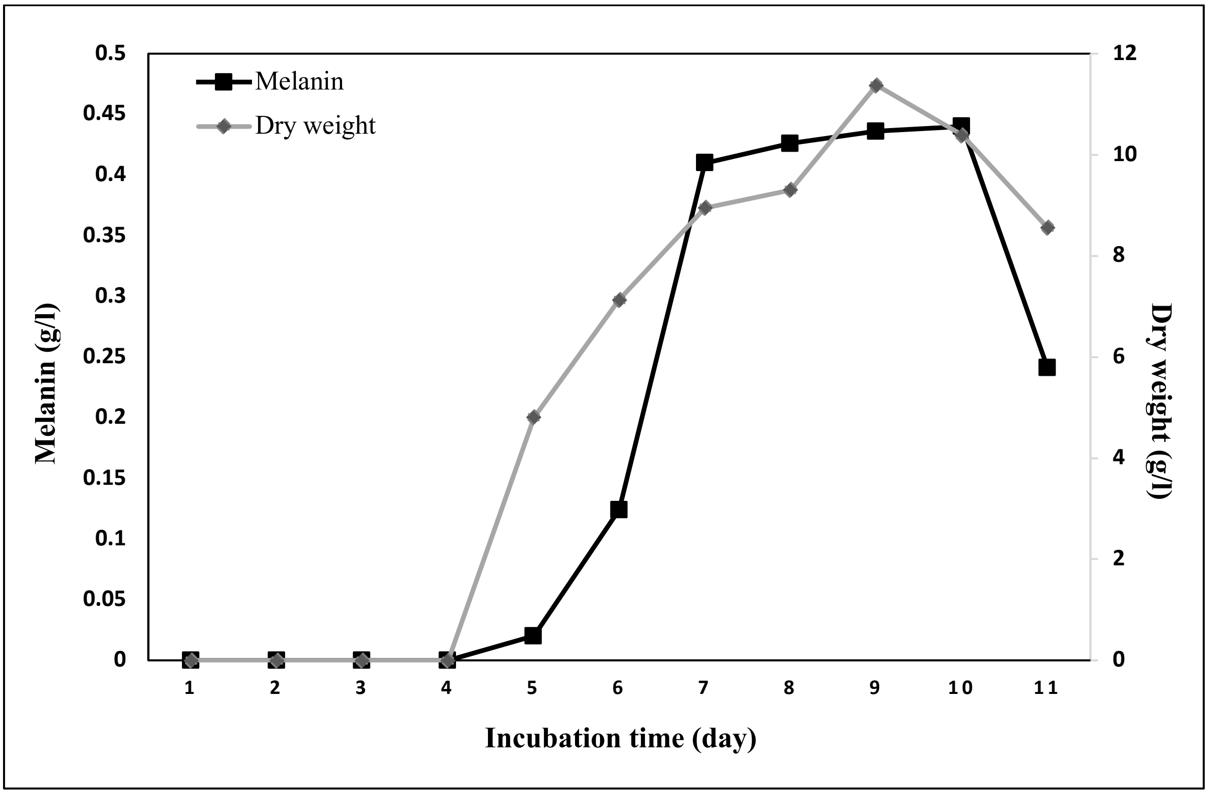

Figure 4. Effect of incubation time on melanin production and dry weight of *H. werneckii* AS1 incubated at 30°C and 180 rpm.

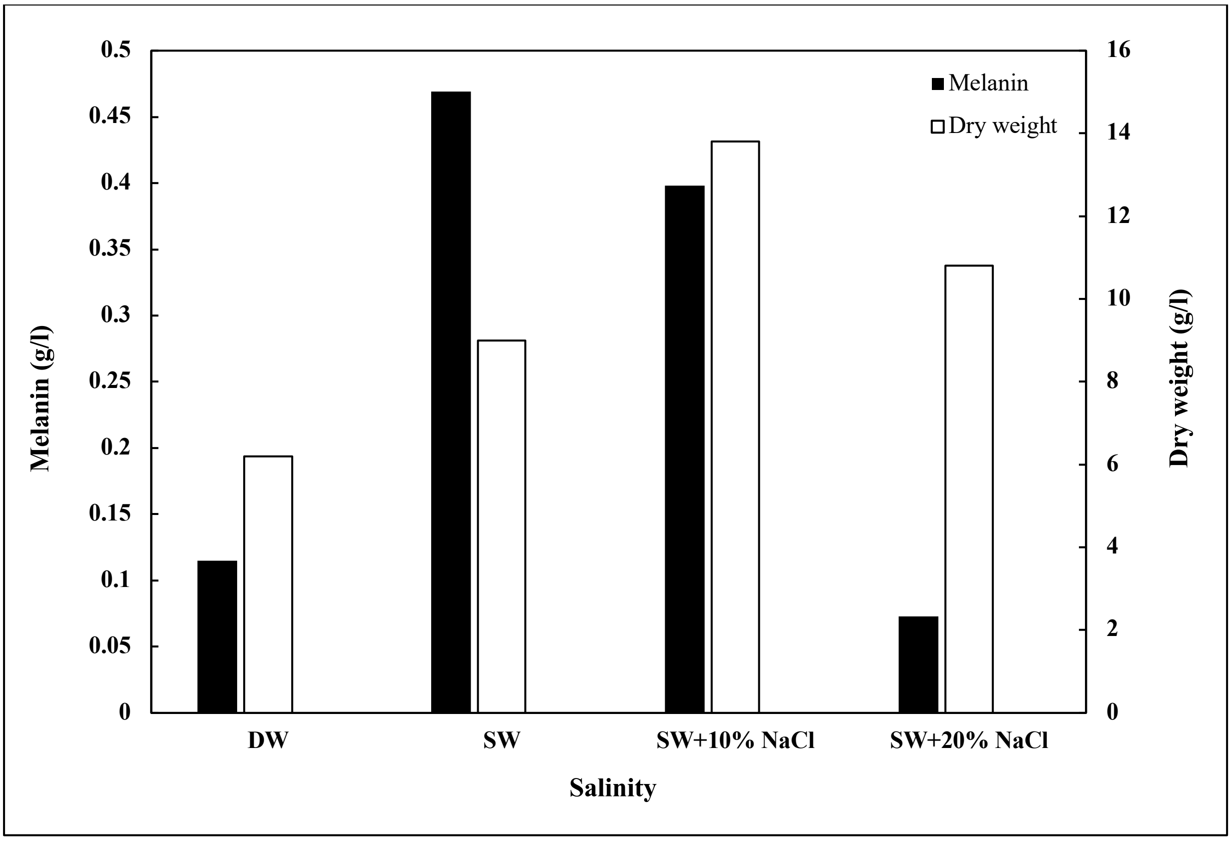

Figure 5. Effect of salinity on melanin production and dry weight of *H. werneckii* AS1 incubated for 10 days at 30°C and 180 rpm.
