# Supplementary material for: Optimization of melanin pigment production from the halotolerant black yeast Hortaea werneckii AS1 isolated from solar salter in Alexandria
Source: BMC Microbiol. 2022 Apr 8;22:92. doi: 10.1186/s12866-022-02505-1 (PMC8991569; doi:10.1186/s12866-022-02505-1)
Supplement: Supplementary file 2 — Additional file 2. [file 12866_2022_2505_MOESM2_ESM.pdf]

> H. werneckii AS1

CCTGCGGAGGGATCATTACTGAGTGTGGGCTCCGGCCCTACCTCCAACCCCATGT
CGAAACGACTCTGTTGCCTCGGGGGCGACCCGGCCTTCGGGCGTCGGGGCCCCC
GGCGGACACCTTCATAACTCTTGCATCTCTTGCGTCTGAGTGATACATATAATCA
ATCAAAACTTTCAACAACGGATCTCTTGGTTCTGGCATCGATGAAGAACGCAGCG
AAATGCGATAAGTAATGTGAATTGCAGAATTCAGTGAATCATCGAATCTTTGAAC
GCACATTGCGCCCCCTGGCATTCCGGGGGGCATGCCTGTTCGAGCGTCATTACAC
CACTCAAGCCTGGCTTGGTATTGAGCGCCGCGGCCTGCCCGCGCGCTCCAATGTC
TCCGGCTGAGCCGTCCGTCTCTAAGCGTTGTGAATAGCGATCGCTTGCGAGGCCC
GGGCGCGCTCGACGCCGTTAAACCCCCCATTTTCTATGGTTGACCTCGGATCAGG
TAGGGATACCCGCTGAACTTAAGCATATCAATAAGCGGAGGA

> H. werneckii AUMC 10270 KX233858.1

TTCCGTAGGGNGACCTGCGGAGGGATCATTACTGAGTGTGGGCTCCGGCCCTACCTCCAACCCCATGT
CG
AAACGACTCTGTTGCCTCGGGGGCGACCCGGCCTTCGGGCGTCGGGGCCCCCGGCGGACACCTTCATA
AC
TCTTGCATCTCTTGCGTCTGAGTGATACATATAATCAATCAAAACTTTCAACAACGGATCTCTTGGTT
CT
GGCATCGATGAAGAACGCAGCGAAATGCGATAAGTAATGTGAATTGCAGAATTCAGTGAATCATCGAA
TC
TTTGAACGCACATTGCGCCCCCTGGCATTCCGGGGGGCATGCCTGTTCGAGCGTCATTACACCACTCA
AG
CCTGGCTTGGTATTGAGCGCCGCGGCCTGCCCGCGCGCTCCAATGTCTCCGGCTGAGCCGTCCGTCTC
TA
AGCGTTGTGAATAGCGATCGCTTGCGAGGCCCGGGCGCGCTCGACGCCGTTAAACCCCCCATTTTCTA
TG
GTTGACCTCGGATCAGGTAGGGATACCCGCTGAACTTAAGCATATCAATAAGCGGAGGAA

> H. werneckii KAS7949 KY659509.1

AGTGAGGTGTTCGGACTGGCCCAGGGAGGTCGGCAACGACCACCCAGGGCCGGAAAGTTCATCAAACT
GA
GTCATTTAGAGGAAGTAAAAGTCGTAACAAGGTCTCCGTAGGTGAACCTGCGGAGGGATCATTACTGA
GT
GTGGGCTCCGGCCCTACCTCCAACCCCATGTCGAAACGACTCTGTTGCCTCGGGGGCGACCCGGCCTT
CG
GGCGTCGGGGCCCCCGGTGGACACCTTCATAACTCTTGCATCTCTTGCGTCTGAGTGATACATATAAT
CA
ATCAAAACTTTCAACAACGGATCTCTTGGTTCTGGCATCGATGAAGAACGCAGCGAAATGCGATAAGT
AA
TGTGAATTGCAGAATTCAGTGAATCATCGAATCTTTGAACGCACATTGCGCCCCCTGGCATTCCGGGG
GG
CATGCCTGTTCGAGCGTCATTACACCACTCAAGCCTGGCTTGGTATTGAGCGCCGCGGCCTGCCCGCG
CG
CTCCAATGTCTCCGGCTGAGCCGTCCGTCTCTAAGCGTTGTGAATAGCGATCGCTTGCGAGGCCCGGG
CG
GCTCGACGCCGTTAAACCCCCCATTTTCTATGGTTGACCTCGGATCAGGTAGGGATACCCGCTGAACT
TA
AGCATATCAATAAGCGGAGGAAAAGAAACCAACAGGGATTGCCCTAGTAACGGCGAGTGAAGCGGCAA
CA
GCTCAAATTTGAAATCTGGCGCAAGCCCGAGTTGTAATTTGTAGAGGATGCTTCTGGGCAGCGGCCGG

TC
TAAGTTCCTTGGAACAGGACGTCATAGAGGGTGAGA

> H. werneckii KAS7942 KY659507.1

AGTGAGGTGTTCGGACTGGCCCAGGGAGGTCGGCAACGACCACCCAGGGCCGGAAAGTTCATCAAACT
GA
GTCATTTAGAGGAAGTAAAAGTCGTAACAAGGTCTCCGTAGGTGAACCTGCGGAGGGATCATTACTGA
GT
GTGGGCTCCGGCCCTACCTCCAACCCCATGTCGAAACGACTCTGTTGCCTCGGGGGCGACCCGGCCTT
CG
GGCGTCGGGGCCCCCGGTGGACACCTTCATAACTCTTGCATCTCTTGCGTCTGAGTGATACATATAAT
CA
ATCAAAACTTTCAACAACGGATCTCTTGGTTCTGGCATCGATGAAGAACGCAGCGAAATGCGATAAGT
AA
TGTGAATTGCAGAATTCAGTGAATCATCGAATCTTTGAACGCACATTGCGCCCCCTGGCATTCCGGGG
GG
CATGCCTGTTCGAGCGTCATTACACCACTCAAGCCTGGCTTGGTATTGAGCGCCGCGGCCTGCCCGCG
CG
CTCCAATGTCTCCGGCTGAGCCGTCCGTCTCTAAGCGTTGTGAATAGCGATCGCTTGCGAGGCCCGGG
CG
GCTCGACGCCGTTAAACCCCCCATTTTCTATGGTTGACCTCGGATCAGGTAGGGATACCCGCTGAACT
TA
AGCATATCAATAAGCGGAGGAAAAGAAACCAACAGGGATTGCCCTAGTAACGGCGAGTGAAGCGGCAA
CA
GCTCAAATTTGAAATCTGGCGCAAGCCCGAGTTGTAATTTGTAGAGGATGCTTCTGGGCAGCGGCCGG
TC
TAAGTTCCTTGGAACAGGACGTCATAGAGGGTGAGAATC

> H. werneckii Hw6 JN997370.1

TCGTAACAAGGTCTCCGTAGGTGAACCTGCGGAGGGATCATTACTGAGTGTGGGCTCCGGCCCTACCT
CC
AACCCCATGTCGAAACGACTCTGTTGCCTCGGGGGCGACCCGGCCTTCGGGCGTCGGGGCCCCCGGCG
GA
CACCTTCATAACTCTTGCATCTCTTGCGTCTGAGTGATACATATAATCAATCAAAACTTTCAACAACG
GA
TCTCTTGGTTCTGGCATCGATGAAGAACGCAGCGAAATGCGATAAGTAATGTGAATTGCAGAATTCAG
TG
AATCATCGAATCTTTGAACGCACATTGCGCCCCCTGGCATTCCGGGGGGCATGCCTGTTCGAGCGTCA
TT
ACACCACTCAAGCCTGGCTTGGTATTGAGCGCCGCGGCCTGCCCGCGCGCTCCAATGTCTCCGGCTGA
GC
CGTCCGTCTCTAAGCGTTGTGAATAGCGATCGCTTGCGAGGCCCGGGCGCGCTCGACGCCGTTAAACC
CC
CCATTTTCTATGGTTGACCTCGGATCAGGTAGGGATACCCGCTGAACTTAAGCATATCAATA

> H. werneckii MCw215 HQ711621.1

TCTCAGCTAGTGACCTGCGGAGGGATCATTACTGAGTGTGGGCTCCGGCCCTACCTCCAACCCCATGT
CG
AAACGACTCTGTTGCCTCGGGGGCGACCCGGCCTTCGGGCGTCGGGGCCCCCGGTGGACACCTTCATA
AC
TCTTGCATCTCTTGCGTCTGAGTGATACATATAATCAATCAAAACTTTCAACAACGGATCTCTTGGTT

CT
GGCATCGATGAAGAACGCAGCGAAATGCGATAAGTAATGTGAATTGCAGAATTCAGTGAATCATCGAA
TC
TTTGAACGCACATTGCGCCCCCTGGCATTCCGGGGGGCATGCCTGTTCGAGCGTCATTACACCACTCA
AG
CCTGGCTTGGTATTGAGCGCCGCGGCCTGCCCGCGCGCTCCAATGTCTCCGGCTGAGCCGTCCGTCTC
TA
AGCGTTGTGAATAGCGATCGCTTGCGAGGCCCGGGCGGTTCGACGCCGTTAAACCCCCCATTTTCTAT
GG
TTGACCTCGGATCAGGTAGGGATACCCGCTGAACTTAAGCATATCAATAAGCGGAGGAAAAGAAACCA
AC
AGGGATTGCCCTAGTAACGGCGAGTGAAGCGGCAACAGCTCAAATTTGAAATCTGGCGCAAGCCCGAG
TT
GTAATTTGTAGAGGATGCTTCTGGGCAGCGGCCGGTCTAAGTTCCTTGGAACAGGACGTCATAGAGGG
TG
AGAATCCCGTATGCGACCGGCTTGCACCCGTCACGTAGCTCCTTCGACGAGTCGAGTTGTTTGGGATG
CA
GCTCTAATGGGAGGTAAATTTCTTCTAAGCTAATACCGGCCAGAGACCGATAGCGCACAGTAGAGTGA
TC
GAAAGATGAAAAGCACTTTGGAAG

> Hortaea sp. F47 FJ755827.1

CTTGGTCATTTAGAGGAAGTAAAAGTCGTAACAAGGTCTCCGTAGGTGAACCTGCGGAGGGATCATTA
CT
GAGTGTGGGCTCCGGCCCTACCTCCAACCCCATGTCGAAACGACTCTGTTGCCTCGGGGGCGACCCGG
CC
TTCGGGCGTCGGGGCCCCCGGTGGACACCTTCATAACTCTTGCATCTCTTGCGTCTGAGTGATACATA
TA
ATCAATCAAAACTTTCAACAACGGATCTCTTGGTTCTGGCATCGATGAAGAACGCAGCGAAATGCGAT
AA
GTAATGTGAATTGCAGAATTCAGTGAATCATCGAATCTTTGAACGCACATTGCGCCCCCTGGCATTCC
GG
GGGGCATGCCTGTTCGAGCGTCATTACACCACTCAAGCCTGGCTTGGTATTGAGCGCCGCGGCCTGCC
CG
CGCGCTCCAATGTCTCCGGCTGAGCCGTCCGTCTCTAAGCGTTGTGAATAGCGATCGCTTGCGAGGCC
CG
GGCGGTTCGACGCCGTTAAACCCCCCATTTTCTATGGTTGACCTCGGATCAGGTAGGGATACCCGCTG
AA
CTTAAGCATATCAATAAGCGGAGGA

> Dothideales sp. LM204 EF060548.1

GAGTCTGGGCCGCACGCGCGCTACACTGACAGAGCCAACGAGTTTTTTTCCTTGGCCGGAAGGTCTGG
GT
AATCTTGTTAAACTCTGTCGTGCTGGGGATAGAGCATTGCAATTATTGCTCTTCAACGAGGAATGCCT
AG
TAAGCGCATGTCATCAGCATGCGTTGATTACGTCCCTGCCCTTTGTACACACCGCCCGTCGCTACTAC
CG
ATTGAATGGCTCAGTGAGGTGTTCGGACTGGCCCAGGGAGGTCGGCAACGACCACCCAGGGCCGGAAA
GT
TCATCAAACTGAGTCATTTAGAGGAAGTAAAAGTCGTAACAAGGTCTCCGTAGGTGAACCTGCGGAGG
GA
TCATTACTGAGTGTGGGCTCCGGCCCTACCTCCAACCCCATGTCGAAACGACTCTGTTGCCTCGGGGG

CG
ACCCGGCCTTCGGGCGTCGGGGCCCCCGGCGGACACCTTCATAACTCTTGCATCTCTTGCGTCTGAGT
GA
TACATATAATCAATCAAAACTTTCAACAACGGATCTCTTGGTTCTGGCATCGATGAAGAACGCAGCGA
AA
TGCGATAAGTAATGTGAATTGCAGAATTCAGTGAATCATCGAATCTTTGAACGCACATTGCGCCCCCT
GG
CATTCCGGGGGGCATGCCTGTTCGAGCGTCATTACACCACTCAAGCCTGGCTTGGTATTGAGCGCCGC
GG
CCTGCCCGCGCGCTCCAATGTCTCCGGCTGACCGTCCGTCTCTAAGCGTTGTGAATAGCGATCGCTTG
CG
AGGCCCGGGCGCGCTCGACGCCGTTAAACCCCCCATTTTCTATGGTTGACCTCGGATCAGGTAGGGGA
TA
CCCGCTGAACTTAAGCATATCATAAGCGGAGGAAAAGAAACCACAGGGATTGCCTAAT

> H. werneckii IFM 4988 AB087199.1

TCCGTAGGTGAACCTGCGGAGGGATCATTACTGAGTGTGGGCTCCGGCCCTACCTCCAACCCCATGTC
GA
AACGACTCTGTTGCCTCGGGGGCGACCCGGCCTTCGGGCGTCGGGGCCCCCGGTGGACACCTTCATAA
CT
CTTGCATCTCTTGCGTCTGAGTGATACATATAATCAATCAAAACTTTCAACAACGGATCTCTTGGTTC
TG
GCATCGATGAAGAACGCAGCGAAATGCGATAAGTAATGTGAATTGCAGAATTCAGTGAATCATCGAAT
CT
TTGAACGCACATTGCGCCCCCTGGCATTCCGGGGGGCATGCCTGTTCGAGCGTCATTACACCACTCAA
GC
CTGGCTTGGTATTGAGCGCCGCGGCCTGCCCGCGCGCTCCAATGTCTCCGGCTGAGCCGTCCGTCTCT
AA
GCGTTGTGAATAGCGATCGCTTGCGAGGCCCGGGCGGTTCGACGCCGTTAAACCCCCCATTTTCTATG
GT
TGACCTCGGATCAGGTAGGGATACCCGCTGAACTTAAGCATATCAATAAGCGGAGGA

> H. werneckii CBS 126986 MH864373.1

CCTTTGTACACACCGCCCGTCGCTACTACCGATTGAATGGCTCAGTGAGGTGTTCGGACTGGCCCAGG
GA
GGTCGGCAACGACCACCCAGGGCCGGAAAGTTCATCAAACTGAGTCATTTAGAGGAAGTAAAAGTCGT
AA
CAAGGTCTCCGTAGGTGAACCTGCGGAGGGATCATTACTGAGTGTGGGCTCCGGCCCTACCTCCAACC
CC
ATGTCGAAACGACTCTGTTGCCTCGGGGGCGACCCGGCCTTCGGGCGTCGGGGCCCCCGGTGGACACC
TT
CATAACTCTTGCATCTCTTGCGTCTGAGTGATACATATAATCAATCAAAACTTTCAACAACGGATCTC
TT
GGTTCTGGCATCGATGAAGAACGCAGCGAAATGCGATAAGTAATGTGAATTGCAGAATTCAGTGAATC
AT
CGAATCTTTGAACGCACATTGCGCCCCCTGGCATTCCGGGGGGCATGCCTGTTCGAGCGTCATTACAC
CA
CTCAAGCCTGGCTTGGTATTGAGCGCCGCGGCCTGCCCGCGCGCTCCAATGTCTCCGGCTGAGCCGTC
CG
TCTCTAAGCGTTGTGAATAGCGATCGCTTGCGAGGCCCGGGCGGTTCGACGCCGTTAAACCCCCCATT
TT
CTATGGTTGACCTCGGATCAGGTAGGGATACCCGCTGAACTTAAGCATATCAATAAGGCGGAGGAA

> H. werneckii JY 54 KM014589.1

TAGGTGAACCTGCGGAGGGATCATTACTGAGTGTGGGCTCCGGCCCTACCTCCAACCCCATGTCGAAA
CG
ACTCTGTTGCCTCGGGGGCGACCCGGCCTTCGGGCGTCGGGGCCCCCGGTGGACACCTTCATAACTCT
TG
CATCTCTTGCGTCTGAGTGATACATATAATCAATCAAAACTTTCAACAACGGATCTCTTGGTTCTGGC
AT
CGATGAAGAACGCAGCGAAATGCGATAAGTAATGTGAATTGCAGAATTCAGTGAATCATCGAATCTTT
GA
ACGCACATTGCGCCCCCTGGCATTCCGGGGGGCATGCCTGTTCGAGCGTCATTACACCACTCAAGCCT
GG
CTTGGTATTGAGCGCCGCGGCCTGCCCGCGCGCTCCAATGTCTCCGGCTGAGCCGTCCGTCTCTAAGC
GT
TGTGAATAGCGATCGCTTGCGAGGCCCGGGCGGTTCGACGCCGTTAAACCCCCCATTTTCTATGGTTG
AC
CTCGGATCAGGTAGGGATACCCGCTGAACTTAAGCATATCAATAAGC

> H. werneckii F5_ITS5 MW509915.1

GCKKACTGMGGAGGGWCATTACTGAGTGTGGGCTCCGGCCCTACCTCCAACCCCATGTCGAAACGACT
CT
GTTGCCTCGGGGGCGACCCGGCCTTCGGGCGTCGGGGCCCCCGGTGGACACCTTCATAACTCTTGCAT
CT
CTTGCGTCTGAGTGATACATATAATCAATCAAAACTTTCAACAACGGATCTCTTGGTTCTGGCATCGA
TG
AAGAACGCAGCGAAATGCGATAAGTAATGTGAATTGCAGAATTCAGTGAATCATCGAATCTTTGAACG
CA
CATTGCGCCCCCTGGCATTCCGGGGGGCATGCCTGTTCGAGCGTCATTACACCACTCAAGCCTGGCTT
GG
TATTGAGCGCCGCGGCCTGCCCGCGCGCTCCAATGTCTCCGGCTGAGCCGTCCGTCTCTAAGCGTTGT
GA
ATAGCGATCGCTTGCGAGGCCCGGGCGGTTCGACGCCGTTAAACCCCCCATTTTCTATGGTTGACCTC
GG
ATCAGGTAGGGATACCCGCTGAACTTAAGCATATCAATAAGCGGAGGAA

> H. werneckii T2 JX141367.1

TCCGTAGGTGAACCTGCGGAGGGATCATTACCGAGTGTGGCGCTCCGGCGCCTCCCTCCAACCCCATG
TC
GAAACGACTCTGTTGCCTCGGGGGCGACCCGGCCTTCGGGCGTCGGGGCCCCCGGCGGACACCTTCAT
AA
CTCTTGCATCTCTTGCGTCTGAGTGATACATATAATCAATCAAAACTTTCAACAACGGATCTCTTGGT
TC
TGGCATCGATGAAGAACGCAGCGAAATGCGATAAGTAATGTGAATTGCAGAATTCAGTGAATCATCGA
AT
CTTTGAACGCACATTGCGCCCCCTGGCATTCCGGGGGGCATGCCTGTTCGAGCGTCATTACACCACTC
AA
GCCTGGCTTGGTATTGAGCGCCGCGGCCTGCCCGCGCGCTCCAATGTCTCCGGCTGAGCCGTCCGTCT
CT
AAGCGTTGTGAATAGCGATCGCTTGCGAGGCCCGGGCGGTTCGACGCCGTTAAACCCCCCATTTTCTA
TG
GTTGACCTCGGATCAGGTAGGGATACCCGCTGAACTTAAGCATATCAATAAGCGGAGGAA

> H. werneckii strain RY 51

CCTGCGGAGGGATCATTACTGAGTGTGGGCTCCGGCCCTACCTCCAACCCCATGTCGAAACGACTCTG
TT
GCCTCGGGGGCGACCCGGCCTTCGGGCGTCGGGGCCCCCGGTGGACACCTTCATAACTCTTGCATCTC
TT
GCGTCTGAGTGATACATATAATCAATCAAAACTTTCAACAACGGATCTCTTGGTTCTGGCATCGATGA
AG
AACGCAGCGAAATGCGATAAGTAATGTGAATTGCAGAATTCAGTGAATCATCGAATCTTTGAACGCAC
AT
TGCGCCCCCTGGCATTCCGGGGGGCATGCCTGTTCGAGCGTCATTACACCACTCAAGCCTGGCTTGGT
AT
TGAGCGCCGCGGCCTGCCCGCGCGCTCCAATGTCTCCGGCTGAGCCGTCCGTCTCTAAGCGTTGTGAA
TA
GCGATCGCTTGCGAGGCCCGGGCGGTTCGACGCCGTTAAACCCCCCATTTTCTATGGTTGACCTCGGA
TC
AGGTAGGGATACCCGCTGAACTTAAGCATATCAATA

> H. werneckii strain Hw5

CCTGCGGAGGGATCATTACTGAGTGTGGGCTCCGGCCCTACCTCCAACCCCATGTCGAAACGACTCTG
TT
GCCTCGGGGGCGACCCGGCCTTCGGGCGTCGGGGCCCCCGGTGGACACCTTCATAACTCTTGCATCTC
TT
GCGTCTGAGTGATACATATAATCAATCAAAACTTTCAACAACGGATCTCTTGGTTCTGGCATCGATGA
AG
AACGCAGCGAAATGCGATAAGTAATGTGAATTGCAGAATTCAGTGAATCATCGAATCTTTGAACGCAC
AT
TGCGCCCCCTGGCATTCCGGGGGGCATGCCTGTTCGAGCGTCATTACACCACTCAAGCCTGGCTTGGT
AT
TGAGCGCCGCGGCCTGCCCGCGCGCTCCAATGTCTCCGGCTGAGCCGTCCGTCTCTAAGCGTTGTGAA
TA
GCGATCGCTTGCGAGGCCCGGGCGGTTCGACGCCGTTAAACCCCCCATTTTCTATGGTTGACCTCGGA
TC
AGGTAGGGATACCCGCTGAACTTAAGCATATCAATA

> Fungal sp. 46 SAB-2015 strain SV671

CATTACTGAGTGTGGGCTCCGGCCCTACCTCCAACCCCATGTCGAAACGACTCTGTTGCCTCGGGGGC
GA
CCCGGCCTTCGGGCGTCGGGGCCCCCGGCGGACACCTTCATAACTCTTGCATCTCTTGCGTCTGAGTG
AT
ACATATAATCAATCAAAACTTTCAACAACGGATCTCTTGGTTCTGGCATCGATGAAGAACGCAGCGAA
AT
GCGATAAGTAATGTGAATTGCAGAATTCAGTGAATCATCGAATCTTTGAACGCACATTGCGCCCCCTG
GC
ATTCCGGGGGGCATGCCTGTTCGAGCGTCATTACACCACTCAAGCCTGGCTTGGTATTGAGCGCCGCG
GC
CTGCCCGCGCGCTCCAATGTCTCCGGCTGAGCCGTCCGTCTCTAAGCGTTGTGAATAGCGATCGCTTG
CG
AGGCCCGGGCGGTTCGACGCCGTTAAACCCCCCATTTTCTATGGTTGACCTCGGATCAGGTAGGGATA
CC
CGCTGAACTTAAGCATATCAATAAGCGGAGGA

> H. werneckii isolate SD 454

TGCGGAGGGATCATTACCGAGTGTGGCGCTCCGGCGCCTCCCTCCAACCCCATGTCGAAACGACTCTG
TT
GCCTCGGGGGCGACCCGGCCTTCGGGCGTCGGGGCCCCCGGCGGACACCTTCATAACTCTTGCATCTC
TT
GCGTCTGAGTGATACATATAATCAATCAAAACTTTCAACAACGGATCTCTTGGTTCTGGCATCGATGA
AG
AACGCAGCGAAATGCGATAAGTAATGTGAATTGCAGAATTCAGTGAATCATCGAATCTTTGAACGCAC
AT
TGCGCCCCCTGGCATTCCGGGGGGCATGCCTGTTCGAGCGTCATTACACCACTCAAGCCTGGCTTGGT
AT
TGAGCGCCGCGGCCTGCCCGCGCGCTCCAATGTCTCCGGCTGAGCCGTCCGTCTCTAAGCGTTGTGAA
TA
GCGATCGCTTGCGAGGCCCGGGCGGTTCGACGCCGTTAAACCCCCCATTTTCTATGGTTGACCTCGGA
TC
AGGTAGGGATACCCGCTGAACTTAAGCATATCAATAAGCGGAGGA

> H. werneckii isolate 002-C7

CCTGCGGAGGGATCATTACCGAGTGTGGCGCTCCGGCGCCTCCCTCCAACCCCATGTCGAAACGACTC
TG
TTGCCTCGGGGGCGACCCGGCCTTCGGGCGTCGGGGCCCCCGGTGGACACCTTCATAACTCTTGCATC
TC
TTGCGTCTGAGTGATACATATAATCAATCAAAACTTTCAACAACGGATCTCTTGGTTCTGGCATCGAT
GA
AGAACGCAGCGAAATGCGATAAGTAATGTGAATTGCAGAATTCAGTGAATCATCGAATCTTTGAACGC
AC
ATTGCGCCCCCTGGCATTCCGGGGGGCATGCCTGTTCGAGCGTCATTACACCACTCAAGCCTGGCTTG
GT
ATTGAGCGCCGCGGCCTGCCCGCGCGCTCCAATGTCTCCGGCTGAGCCGTCCGTCTCTAAGCGTTGTG
AA
TAGCGATCGCTTGCGAGGCCCGGGCGGTTCGACGCCGTTAAACCCCCCATTTTCTATGGTTGACCTCG
GA
TCAGGTAGGGATACCCGCTGAACTTAAGCATATCAATAAGCGGAGGA

> H. werneckii strain ATCC 36317

TGAGTGTGGGCTCCGGCCCTACCTCCAACCCCATGTCGAAACGACTCTGTTGCCTCGGGGGCGACCCG
GC
CTTCGGGCGTCGGGGCCCCCGGTGGACACCTTCATAACTCTTGCATCTCTTGCGTCTGAGTGATACAT
AT
AATCAATCAAAACTTTCAACAACGGATCTCTTGGTTCTGGCATCGATGAAGAACGCAGCGAAATGCGA
TA
AGTAATGTGAATTGCAGAATTCAGTGAATCATCGAATCTTTGAACGCACATTGCGCCCCCTGGCATTC
CG
GGGGGCATGCCTGTTCGAGCGTCATTACACCACTCAAGCCTGGCTTGGTATTGAGCGCCGCGGCCTGC
CC
GCGCGCTCCAATGTCTCCGGCTGAGCCGTCCGTCTCTAAGCGTTGTGAATAGCGATCGCTTGCGAGGC
CC
GGGCGGTTCGACGCCGTTAAACCCCCCATTTTCTATGGTTGACCTCGGATCAGGTAGGGATACCCGCT
GA
ACTTAAGCATATCAATAAGCGGAGGA
